# Programmable CAR and antigen pairing for solid tumor immunotherapy

**DOI:** 10.1016/j.omton.2026.201296

**Published:** 2026-08-03

**Authors:** Ziliang Huang, Yingxiao Wang

**Affiliations:** 1 State Key Laboratory of Ultrasound in Medicine and Engineering, College of Biomedical Engineering, Chongqing Medical University, Chongqing 400016, China; 2 Alfred E. Mann Department of Biomedical Engineering, University of Southern California, 1042 Downey Way, Los Angeles, CA 90089, USA; 3 Shu Chien-Gene Lay Department of Bioengineering, Institute of Engineering in Medicine, University of California, San Diego, 9500 Gilman Drive, La Jolla, CA 92093, USA

## Main text

In our recent publication in Nature Communications, “*Engineering programmable CAR and antigen pairing via drug-gated light activation*,” we developed a modular CAR-T platform designed to address two central barriers in solid-tumor immunotherapy: antigen escape and on-target/off-tumor toxicity.^1^ While CAR-T therapies have achieved remarkable success in hematologic malignancies, their efficacy in solid tumors remains limited by heterogeneous antigen expression, dynamic antigen loss, and the difficulty of identifying targets that are both broadly expressed on tumors and absent from normal tissues. We reasoned that a more flexible CAR-T system should be able to retarget different tumor antigens without redesigning the CAR itself, while also restricting CAR activity to local tumor regions to improve safety.^2^

To achieve programmable antigen targeting, we engineered a PEbody-based programmable antigen-targeting CAR (PEbody PAT CAR). PEbody is a monobody derived from the human fibronectin type III domain that specifically binds R-phycoerythrin (PE).^3^ By using PE-conjugated antibodies as adapters, the same PEbody CAR-T cells can be redirected toward user-defined antigens simply by switching the antibody. This design allows the sequential or simultaneous targeting of multiple antigens, offering a practical strategy to respond to antigen escape or tumor heterogeneity without generating a new CAR-T product for each target.^4^

We first validated the flexibility of this PEbody PAT CAR platform *in vitro*. PEbody CAR-T cells could be programmed to kill tumor cells expressing different antigens, including CD19, CD20, CD38, EGFR, MUC1, HER2, PD-L1, and PSMA, depending on the PE-conjugated antibody provided. Importantly, the same CAR-T cells could be redirected from CD19 to CD20 when CD19 antigen escape occurred in a B cell acute lymphoblastic leukemia model.^5^ In heterogeneous tumor models, the simultaneous use of multiple PE-conjugated antibodies achieved more comprehensive tumor killing than single-antigen targeting.^6^ These findings established that PEbody CAR-T cells can function as a reusable and retargetable CAR-T platform.

However, expanding antigen targetability also increases the risk of off-tumor toxicity, particularly when targeting antigens that are not strictly tumor specific. To address this safety challenge, we integrated our drug-gated light-activation system (DGLA), based on tamoxifen-gated photoactivatable Cre.^7^ In this design, CAR expression requires both tamoxifen priming and local blue-light stimulation, creating an AND-gated control mechanism. We showed that DGLA could tightly regulate reporter expression, conventional CD19 CAR expression, as well as PEbody PAT CAR expression in T cells. Functionally, DGLA-PAT CAR-T cells displayed light- and drug-dependent activation and cytotoxicity, allowing antigen-programmable killing to be spatially confined.^8^

**Figure 1:**
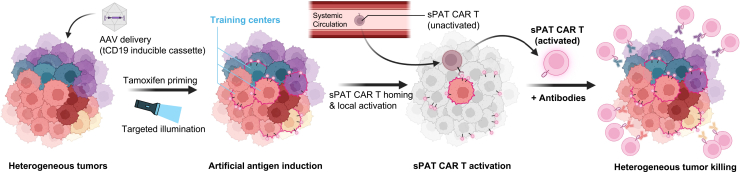
Schematic mechanism of DGLA-sPAT CAR-T therapy enabling spatially restricted activation and broad elimination of heterogeneous solid tumors Heterogeneous tumors are first transduced by AAV carrying a DGLA-controlled inducible tCD19 cassette. After tamoxifen priming, targeted blue-light illumination induces artificial tCD19 antigen expression only within the illuminated tumor region, generating local “training centers.” Systemically delivered anti-CD19 synNotch sPAT CAR-T cells home to the tumor and are locally activated by tCD19-positive training-center cells, triggering expression of the PEbody programmable antigen-targeting CAR. In the presence of PE-conjugated antibodies, activated sPAT CAR-T cells are redirected toward broadly expressed tumor antigens, enabling killing across heterogeneous tumor cell populations while spatially restricting CAR-T cytotoxicity to targeted tumor regions. Created in BioRender https://BioRender.com/cmrdkfa.

We then extended this strategy beyond direct CAR induction and developed DGLA-sPAT, a programmable CAR-antigen pairing system (Figure 1). In this approach, local tumor cells are engineered or “vaccinated” to express a clinically validated synthetic antigen, such as truncated CD19 (tCD19),^9^ under DGLA control. These induced tumor cells act as local “training centers” for systemically delivered synNotch-controlled PAT (sPAT) CAR-T cells. Upon recognizing induced tCD19 through an anti-CD19 synNotch receptor, the T cells express the PEbody PAT CAR and can then be guided by PE-conjugated antibodies to kill the broader tumor population through endogenous tumor-associated antigens such as PD-L1 or PSMA. This design decouples the antigen used for local T cell activation from the antigen used for tumor killing.

A key advantage of this “training center” strategy is that only a subpopulation of tumor cells needs to be induced to activate therapeutic T cells locally. Once trained, the sPAT CAR-T cells can eliminate neighboring tumor cells that do not express the induced antigen, as long as they express the antibody-targeted tumor-associated antigen. *In vitro*, DGLA-sPAT mediated robust killing of MDA-MB-231 and PC-3 tumor models, even when light-induced antigen expression was partial. This suggested that the system could tolerate incomplete gene delivery or heterogeneous induction, both of which are likely in practical therapeutic settings.

We further evaluated the system *in vivo* using bilateral tumor models. DGLA enabled local gene activation only in illuminated tumors after tamoxifen priming. Systemically administered sPAT CAR-T cells preferentially homed to and became activated at tumors expressing the synthetic tCD19 antigen, while control tumors lacking tCD19 showed minimal T cell activation. When PE-conjugated antibodies were administered, tumor suppression occurred selectively at the locally primed tumor site, whereas the contralateral tumor, used to model antigen-positive normal tissue, was largely spared.

To better approximate clinical delivery, we also tested *in situ* tumor vaccination using adeno-associated virus (AAV) vectors encoding the DGLA and inducible tCD19 modules. Even in this setting, local light activation of AAV-delivered DGLA induced tumor antigen presentation, recruited and activated sPAT CAR-T cells, and produced significant tumor suppression. Importantly, the unilluminated contralateral tumors continued to grow, supporting the spatial specificity of the approach. Similar results in both triple-negative breast cancer and prostate cancer models further demonstrated the generalizability of DGLA-sPAT across tumor types and target antigens.

Finally, we directly compared DGLA-sPAT with conventional constitutive PSMA CAR-T therapy in a model designed to evaluate on-target/off-tumor toxicity, where PSMA was expressed both in tumor cells and in the liver.^10^ Although both approaches controlled tumor growth, conventional PSMA CAR-T cells caused systemic toxicity, including weight loss, elevated cytokines, increased serum alanine aminotransferase, and liver immune infiltration and tissue damage. In contrast, DGLA-sPAT CAR-T therapy achieved tumor control while avoiding detectable liver toxicity, supporting the premise that local training and spatially confined CAR activation can improve the therapeutic window.

In summary, our study establishes a programmable and spatially controlled CAR-T framework that integrates three layers of engineering: PEbody-based antibody-programmable antigen recognition, drug-gated light control of local antigen presentation, and synNotch-mediated CAR induction. This system enables flexible targeting of heterogeneous tumors while restricting CAR-T activation to defined tumor regions. More broadly, DGLA-sPAT provides a general strategy for decoupling CAR design from antigen selection and for converting selected tumor sites into local immune-training centers. This approach may be particularly useful for clinically accessible, locally advanced or unresectable solid tumors where regional control can be leveraged to improve both efficacy and safety.

## Acknowledgments

This work is supported by grants from 10.13039/100000002NIH HL121365, CA262815, CA279813, EB029122, GM140929, and HD107206 (Y.W.). This work was also supported by the GT3 Core Facility of the 10.13039/100018259Salk Institute with funding from NIH-10.13039/100000054NCI CCSG: P30 014195, an 10.13039/100000065NINDS R24 Core Grant and funding from 10.13039/100000053NEI.

## Declaration of interests

Y.W. is a scientific co-founder and consultant of Cell E&G Inc. and Acoustic Cell Therapy Inc.
